# Impact of a Pharmacy-Led Transition of Care Service on Post-Discharge Medication Adherence

**DOI:** 10.3390/pharmacy7030128

**Published:** 2019-08-31

**Authors:** Alaina Stroud, Georges Adunlin, Jessica W. Skelley

**Affiliations:** 1 Department of Pharmacy Practice, Samford University McWhorter School of Pharmacy, Birmingham, AL 35229, USA; 2 Department of Pharmaceutical, Social and Administrative Sciences, Samford University McWhorter School of Pharmacy, 800 Lakeshore Drive, Birmingham, AL 35229, USA

**Keywords:** transition of care, adherence, discharge medications

## Abstract

This study assesses the effectiveness of a pharmacy-led transition of care (TOC) service on increasing patients’ understanding of, and reported adherence to, medication post hospital discharge. A cross-sectional survey was administered to patients who were discharged from the hospital with at least one medication received via bedside delivery from the TOC service. Adherence was assessed by asking the patient if they had taken their discharge medications as instructed by the prescriber. Satisfaction with the discharge medication counseling service was assessed through a five-point Likert scale. Descriptive statistics were conducted for all questionnaire items and qualitative data was examined using content analysis. The majority of patients (73%) were counseled on their medication(s) before leaving the hospital. Among those who received counseling, 76 patients had a better understanding of their medication(s). Ninety-five percent of the patients reported adherence, and all six of the patients reporting non-adherence claimed they were not counseled on their medications prior to discharge. Many patients had questions regarding their medication during the follow-up phone call, substantiating the need for further follow-up with patients once they have left the hospital environment. The implementation of medication bedside delivery and counseling services, followed by outpatient adherence monitoring via a transitional care management service, can result in higher levels of reported medication adherence.

## 1. Introduction

Medication non-adherence is an important health care consideration that largely affects health outcomes as well as health care costs [1,2,3,4]. A 2014 study predicted that between $100–300 billion of avoidable health care costs can be credited to non-adherence, suggesting that interventions that increase medication adherence would improve both health and financial outcomes [5]. While medication adherence is a challenge in all care settings, it can be particularly problematic for patients transitioning from an inpatient to outpatient setting following a hospital discharge, due to a variety of factors including changes in health status and new or adjusted medication lists [6,7]. One potential means to improve adherence during care transitions is through a transition of care (TOC) service. In the inpatient health care setting, education about medications often occurs at the time of patient discharge. Despite educational intervention, evidence still points to high rates of medication errors, adverse drug events, and patient nonadherence that occur following patient discharge from the hospital [8].

In recent years, pharmacists have taken a more active role in TOC services and are demonstrating a positive impact on clinical outcomes [9]. Studies have sought to define the role of the pharmacist in TOC services and have focused primarily on outcomes such as readmission rates, mortality, and emergency department visits [8,10]. However, there is currently a lack of published data on other outcomes of bedside medication delivery programs, such as adherence. The purpose of this study was to address the research–practice gap by focusing primarily on medication adherence and patient satisfaction outcomes of a pharmacist-led TOC service that includes medication bedside delivery and telephone follow-up.

Proxsys Rx, LLC is a pharmacy management company that partners with hospitals and health systems to fill pharmacy gaps that may exist. Proxsys Rx operates a bedside medication delivery service inside these health systems, in hopes of decreasing patient burden of having to obtain their medications from an outside pharmacy and improving outcomes. The service is a free, technology-enabled program that allows patients to receive their medications before going home. Patients have the option to opt-out if they do not wish to participate. Once a patient is enrolled in the service, all discharge medications will be sent to the hospital’s outpatient pharmacy to be filled. Once the medications are filled, a member of the pharmacy team will deliver the medication to the patient’s room where they will ask the patient if they would like to be counseled on their medication by a pharmacist. Patients who are discharged with at least one medication via the bedside delivery service are placed on a call list to be contacted within 48–72 h (2–3 business days) for follow-up regarding their medication(s). Follow-up phone calls are typically made by a pharmacist, pharmacy intern, or other persons under the direct supervision of a pharmacist. For the purposes of this study, all phone calls were made by a fourth-year pharmacy intern. Eight hospitals (three in Alabama and five in Mississippi) were involved in this study.

The primary outcome of this study is adherence, as reported by the patient during the post-discharge follow-up phone call (Appendix A, question 4). The secondary outcomes are understanding of medication administration after discharge, patient satisfaction with the bedside delivery service measured via a five-point Likert scale, and the need for further counseling or instruction during the follow-up phone call, which was demonstrated with the final open-ended survey question.

## 2. Materials and Methods

A seven-item, cross-sectional survey (Appendix A) was developed and administered over the course of three months to consenting patients. As part of a Proxsys Rx, LLC routine follow-up, patients were contacted within two to three business days after discharge. In order to validate this tool, the authors pre-tested the survey using a convenience sample of ten individuals with a variety of educational backgrounds. Minor revisions were made to the initial wording of the survey based on respondents’ feedback. Patients were eligible to receive the survey if they were 18 years or older, had recently been discharged with at least one medication from a hospital in Alabama or Mississippi whose pharmacy is owned and operated by Proxsys Rx, and if they gave consent to participate in the telephonic survey during their follow-up call. The purpose of the phone call was to ensure that the patients had received their medication(s) from the hospital, were taking the medication(s) as prescribed, were not experiencing any serious side effects, and did not have any questions or concerns regarding the medication(s). Adherence was assessed by asking the patient if they had taken their discharge medications as instructed by the prescriber. Satisfaction with the discharge medication counseling service was assessed through a five-point Likert scale, with a maximum score of five indicating complete satisfaction and a minimum score of one meaning the patient is very dissatisfied with the service. 

Descriptive statistics were conducted for all questionnaire items, with proportions and mean plus or minus standard deviation, summarized as appropriate. The chi-square test was used for comparisons involving categorical variables, except when the expected cell size fell below five, in which case the Fisher’s exact test was used. Comparisons were considered statistically significant at the five percent level. Qualitative data was also examined using content analysis. Collected data was entered into Excel documents on a protected drive and was only available to the investigators. All analyses were performed using Stata (Version 13.0; Stata Corp., College Station, TX, USA). All patients gave their informed consent for inclusion before they participated in the study. The study was conducted in accordance with the Declaration of Helsinki, and the protocol was approved by the Samford University Institutional Review Board of EXMT-P-18-S-12.

## 3. Results

### 3.1. Descriptive Analysis

A total of 300 patients were contacted over the course of three months, and out of this group, 108 patients completed the survey. There were some differences in the baseline characteristics (Table 1). The majority of the participants were women (*n* = 75), 45 to 65 years of age (*n* = 45) and had a college degree of higher (*n* = 37). The post-discharge medications were new for the majority of the study participants (*n* = 59). The majority of patients also indicated that they had been counseled on their medications prior to discharge (*n* = 78). Of these patients, 91% stated that the counseling provided them with a better understanding of their medications and 100% reported adherence. Of the total 108 patients, 95% reported adherence, regardless of counseling prior to discharge. One-hundred patients indicated that they were very satisfied with the TOC services provided, while 7 patients said they were satisfied, and only 1 patient stated that they were neither satisfied nor dissatisfied. See Table 2 for a complete description of results.

As shown in Table 1, because of the relatively homogenous nature of the study sample, it was methodologically impractical to perform bivariate analyses on several variables, given the small number of participants across nondominant categories including: satisfaction level with counseling, previous medication use, and medication adherence.

Table 2 also further divides the data into those patients that did receive counseling prior to discharge, versus those that did not. Of note, the group that received counseling had a higher percentage of patients that were adherent to their medications and were “very satisfied” with the service. 

### 3.2. Content Analysis

Several patients (*n =* 35, 32%) had questions or comments regarding their medications during the follow-up phone call. Subthemes that emerged from reviewing the open-ended question, which gave the respondent an opportunity to provide any additional information, included: patients not remembering being counseled or someone being counseled on their behalf, concerns regarding adverse drug reactions, needing further instruction on how to take the medications, and others. We provided a few specific examples of comments that were categorized into the subthemes listed below:

1. Patient did not remember being counseled or a family member was counseled on their behalf (*n* = 17).
  “I was probably counseled but don’t really remember because I was groggy from surgery.”
  “They may have gone over the medications with my sister, but I don’t remember.”
  “I had just come out of surgery, so I do not remember anyone counseling on the medication.”
  “I was coming off the anesthesia, so my husband was counseled but not me directly.”
  “I was counseled but was very groggy, so I do not remember it well.”
  “I do not remember being counseled but may have been.”
  “I may have been counseled, don’t really remember. I was pretty out of it.”
  “My wife was counseled on the medications because I was still unconscious.”
2. Questions or comments regarding adverse drug reactions (*n* = 8).
  “What kind of antibiotic is this? It makes me feel really dizzy.”
  “I do not like the way the hydrocodone makes me feel.”
  “One makes me pee a lot during the night.”
  “I am having trouble going to the bathroom. I have not gone in 3 days and was wondering if it has to do with the medications? I was counseled on the medications, but no one explained this side effect to me.”
  “I stopped taking the medication because I did not like the way it made me feel.”
  “The medications are making me nauseous.”
3. Patients needing further instructions on how to take their medication (*n* = 7).
  “I stopped taking the medications because I took them an hour apart on the first day and it made me feel like I was going to fall out or have a seizure.”
  “I was not counseled on how or when to take them.”
  “Am I supposed to wait to take the bladder spasm medication when I started to feel a spasm/leakage coming on, or should I be taking it regularly?”
  “My wife was counseled because I was out of it. I have not needed to take any of the medications.”
  “I am confused on how to take the carafate, so I am not sure if I have been taking it accurately.”
  “How often am I supposed to apply the cream? The nurse gave it to me, but it did not come with any directions.”
4. Other questions or comments (*n* = 3).
  “I am concerned about a possible infection. I was not given any antibiotics after the procedure to prevent infection and the spot is getting really red.”
  “I am taking the pain meds every four hours, but pain still persists. Is there anything I can take to help with this? I was told ibuprofen 400 mg was fine to take in between doses.”
  “I have a stomach virus, and it is not getting any better. I am still going to the bathroom just as much as before I went to the hospital.”

All patients included in subtheme 1 were among those who were not counseled on their medications prior to discharge. Interestingly, only 4 of the 18 patients in subthemes 2–4 reported not being counseled on their medications.

Eighty percent of the patients who were deemed “non-adherent” based on their reports had questions and/or concerns regarding their medications, and 100% of the patients deemed non-adherent claimed that they were not counseled on their medications prior to discharge. It may also be important to note that 80% of those reporting non-adherence were discharged with multiple medications.

All patients who had questions and/or concerns rated their satisfaction as “very satisfied.” This is likely due to the fact that these patients greatly appreciated the follow-up phone call, in which the authors were able to address their questions or concerns.

## 4. Discussion

The purpose of this study was to assess the effectiveness of a pharmacy-led TOC service in increasing patients’ adherence to and understanding of medication post hospital discharge. It is already known that care transitions lead to an increased risk of medication errors that can result in adverse events, prolonged hospital admissions, earlier readmissions, and overutilization of health care resources [11]. While pharmacist intervention during and after hospitalization has been frequently studied in the areas of medication reconciliation and discharge counseling, or on the use of a follow-up phone call, this is one of the few studies that have assessed medication adherence post-discharge in a program that includes all of these factors alongside bedside delivery of medications [12,13,14,15,16,17,18,19].

The findings of this study illustrate the potential benefit of a TOC service that includes bedside delivery of medication, pre-discharge medication counseling, and a post-discharge follow-up phone call within two to three business days. The study found that patients are more likely to be adherent if they were counseled on their medications prior to discharge and if they were more satisfied with the overall service provided. While it was not directly studied, it is likely that even the bedside delivery of medications alone may contribute greatly to post-discharge medication adherence, as many patients may not be able to pick up their medications from somewhere else in a timely manner after leaving the hospital. The authors also felt that it was significant to point out the findings of the open-ended survey question which revealed that 32% of patients still had questions or concerns regarding their medications, regardless of being counseled prior to discharge. This highlights the importance of a post-discharge follow-up phone call to patients leaving the hospital with medications.

While the current study provides great insight into the impact of a TOC service implemented by Proxsys Rx, there are several limitations that need to be highlighted. First, adherence was determined on the basis of patient self-report. Ideally, medication adherence would be confirmed with additional data points such as prescription filling history; or, in the case of this study, it may have been helpful to have a pill count or at least ask additional open-ended questions in regard to how the patient is taking their medications. By relying on patient self-reported adherence, rates of adherence could potentially be overestimated. Patients were not questioned as to the manner of how they were using their medications to ensure that they were in fact using them appropriately, which may have further identified cases of adherence or unintended non-adherence. Additionally, because the study included administration of a survey during the telephone follow-up to assess medication adherence and patient satisfaction, the results could be affected by patients’ social desirability bias when answering the questionnaire. The telephonic survey could only be administered if the phone call was answered and if the caller spoke directly to the patient. Oftentimes, even when the phone was answered, it was a family member or caregiver speaking on the patient’s behalf. This likely contributed to the small study population. A face-to-face, or even an electronically distributed questionnaire, may have generated a greater response rate and should be taken into consideration for future studies. Face-to-face interaction could also inform the researchers on other issues the patient may be experiencing that are less detectable via telephone [19]. Specifically, respondents may be more willing to give longer periods of time to respond to the questions in a face-to-face situation rather than over the phone. The interviewer is also given the opportunity to explain and probe out questions that may help uncover other information that the participant may not necessarily be willing to share over the phone. Additionally, the study only looked at the short-term impact of the TOC service. This likely contributed to the higher than normal reported adherence rate. Future directions include developing a randomized clinical trial, in which patients utilizing the TOC service are compared to those who are not utilizing the TOC service and assessing intermediate and long-term outcomes of the service using clinical records (i.e., readmission rates).

## 5. Conclusions

Patients who received medication counseling before being discharged, and who were more satisfied with their medication counseling experience, were more likely to be adherent to their medication(s). The implementation of medication bedside delivery and counseling services, followed by adherence monitoring into practical patient care, may result in an improvement of medication adherence. Further studies are needed to investigate the frequency at which such an intervention needs to be delivered, determine their cost-effectiveness, and establish whether they can lead to better health outcomes for patients.

## Figures and Tables

**Table 1:** Patient demographics.

Patient Characteristics	Frequency (N = 108)	%
*Gender*		
Male	33	30.56
Female	75	69.44
*Age* (*years*)		
<44	36	33.64
45 to 64 years	45	42.06
≥65	26	24.30
*Educational level*		
No High School	17	15.74
High School Diploma	24	22.22
Some College	30	27.78
College degree and Higher	37	34.26
*Hospital Type*		
Church-operated	18	16.67
Public	22	20.37
Privately owned	68	62.96
*Number of Hospital Beds*		
<199	20	18.52
200 to 399	54	50
≥400	34	31.48

**Table 2:** Medication characteristics and study results.

Patient Characteristics	Frequency for All Patients (N = 108) *n* (%)	Patients Who Received Counseling (N = 78) *n* (%)	Patients Who Did Not Receive Counseling (N = 30) *n* (%)
*Medication Type* ^1^			
Pain (NSAID/Opioid)	87 (80.55)	67 (85.89)	22 (73.33)
Anticoagulant	24 (22.22)	19 (24.36)	5 (16.67)
Cardiac	13 (12.03)	11 (14.10)	2 (6.67)
Antibiotics	15 (13.88)	12 (15.38)	3 (10)
Antiemetic	26 (24.07)	21 (26.92)	6 (20)
*New Medication Use*			
Yes	49 (45.37)	35 (44.87)	15 (50)
No	59 (54.63)	43 (55.13)	15 (50)
*Counseling Experience*			
Yes	78 (72.22)	78 (100)	0
No	30 (27.78)	0	30 (100)
*Understanding of Medication Administration* ^2^			
Yes	71 (91.03)	76 (97.44)	N/A
No	7 (8.97)	2 (2.56)	N/A
*Satisfaction Level*			
Very satisfied	100 (92.59)	74 (94.87)	25 (83.33)
Satisfied	7 (6.48)	4 (5.13)	4 (13.33)
Neither satisfied nor dissatisfied	1 (0.93)	0	1 (3.33)
Dissatisfied	0	0	0
Very dissatisfied	0	0	0
*Medication Adherence*			
Yes	103 (95.37)	78 (100)	25 (83.33)
No	5 (4.63)	0	5 (16.67)

^1^ Numbers exceed N and percentage does not come to 100%, because some patients are taking more than one medication. ^2^ Patients were only asked this question if their response to the previous question was “yes”.

## Appendix A

Consent Statement

Do you consent to allowing your responses to be used as a part of a study to help us improve our pharmacy services? (YES/NO).

1. Have you taken this medication before? (YES/NO)
2. Did you find it helpful for the pharmacy staff to deliver your medications to you before leaving the hospital? (YES/NO)
3. Were you counseled on the medications before leaving the hospital? (YES/NO)
  - If YES to #3—After being counseled on the medications, did you have a better understanding of your medications (i.e., how to take them and what they are for)? (YES/NO)
4. Have you been taking the medications that you were sent home with as your doctor instructed? (YES/NO)
5. Overall, how would you rate your satisfaction with the pharmacy services, the options being:
  - Very satisfied
  - Somewhat satisfied
  - Neither satisfied nor dissatisfied
  - Somewhat dissatisfied
  - Very dissatisfied
6. One last question, what is the highest grade or level of school you have completed?
  - Did Not Complete High School
  - High school diploma
  - Some College
  - College degree
  - Advanced Graduate work or Ph.D.
7. Do you have any questions or concerns regarding these medications?

## References

[1] Mojtabai R., Olfson M. (2003). Medication costs, adherence, and health outcomes among Medicare beneficiaries. Health Aff..

[2] Sokol M.C., McGuigan K.A., Verbrugge R.R., Epstein R.S. (2005). Impact of medication adherence on hospitalization risk and healthcare cost. Med. Care.

[3] Kripalani S., Yao X., Haynes R.B. (2007). Interventions to enhance medication adherence in chronic medical conditions: A systematic review. Arch. Intern. Med..

[4] Gellad W.F., Grenard J.L., Marcum Z.A. (2011). A Systematic Review of Barriers to Medication Adherence in the Elderly: Looking Beyond Cost and Regimen Complexity. Am. J. Geriatr. Pharmacother..

[5] Iuga A.O., McGuire M.J. (2014). Adherence and health care costs. Risk Manag. Health Policy.

[6] Forster A.J., Murff H.J., Peterson J.F., Gandhi T.K., Bates D.W. (2003). The Incidence and Severity of Adverse Events Affecting Patients after Discharge from the Hospital. Ann. Intern. Med..

[7] Mulhem E., Lick D., Varughese J., Barton E., Ripley T., Haveman J. (2013). Adherence to Medications after Hospital Discharge in the Elderly. Int. J. Fam. Med..

[8] Ensing H.T., Stuijt C.C., Bemt B.J.V.D., Van Dooren A.A., Karapinar-Çarkit F., Koster E.S., Bouvy M.L. (2015). Identifying the Optimal Role for Pharmacists in Care Transitions: A Systematic Review. J. Manag. Care Spéc. Pharm..

[9] Mueller S.K., Sponsler K.C., Kripalani S., Schnipper J.L. (2012). Hospital-based Medication Reconciliation Practices: A Systematic Review. Arch. Intern. Med..

[10] Lam S.W., Sokn E. (2019). Effect of Pharmacy-Driven Bedside Discharge Medication Delivery Program on Day 30 Hospital Readmission. J. Pharm. Pract..

[11] Splawski J., Minger H. (2016). Value of the Pharmacist in the Medication Reconciliation Process. Pharm. Ther..

[12] Tran T., Khattar S., Vu T.T., Potter M., Hodding J., Kuo G.M., Le J. (2017). Impact of pharmacist discharge counseling on hospital readmission and emergency department visit. J. Hosp. Adm..

[13] Mekonnen A.B., McLachlan A.J., Brien J.E. (2016). Pharmacy-led medication reconciliation programmes at hospital transitions: A systematic review and meta-analysis. J. Clin. Pharm. Ther..

[14] Odegard P.S., Carpinito G., Christensen D.B. (2013). Medication adherence program: Adherence challenges and interventions in type 2 diabetes. J. Am. Pharm. Assoc..

[15] Odegard P.S., Christensen D.B. (2013). MAP study: RCT of a medication adherence program for patients with type 2 diabetes. J. Am. Pharm. Assoc..

[16] Odeh M., Scullin C., Fleming G., Scott M.G., Horne R., McElnay J.C. (2019). Ensuring continuity of patient care across the healthcare interface: Telephone follow-up post-hospitalization. Br. J. Clin. Pharmacol..

[17] Gilmore V., Efird L., Fu D., LeBlanc Y., Nesbit T., Swarthout M. (2015). Implementation of transitions-of-care services through acute care and outpatient pharmacy collaboration. Am. J. Health Syst. Pharm..

[18] Spiegel B., Shane R., Palmer K., Luong D.O. (2018). Cost-effectiveness of pharmacist postdischarge follow-up to prevent medication-related admissions. Am. J. Acc. Care.

[19] Tiene D. (2000). Online discussions: A survey of advantages and disadvantages compared to face-to-face discussions. J. Educ. Multimed. Hypermedia.

